# Impact of interventions at frequently used suicide locations on occurrence of suicides at other sites: a systematic review and meta-analysis

**DOI:** 10.1017/S0033291725100792

**Published:** 2025-06-24

**Authors:** Lay San Too, Sangsoo Shin, Yamna Taouk, Jane Pirkis, Mark Sinyor, Paul Siu Fai Yip, Keith Hawton

**Affiliations:** 1 Centre for Mental Health and Community Wellbeing, Melbourne School of Population and Global Health, The University of Melbourne, Parkville, VIC, Australia; 2 Centre for Health Equity, Melbourne School of Population and Global Health, The University of Melbourne, Parkville, VIC, Australia; 3 Department of Psychiatry, Sunnybrook Health Sciences Centre, University of Toronto, Toronto, ON, Canada; 4 Hong Kong Jockey Club Centre for Suicide Research and Prevention, The University of Hong Kong, Pokfulam, Hong Kong SAR; 5 Centre for Suicide Research, University of Oxford, Oxford, UK

**Keywords:** frequently used suicide location, means restriction, meta-analysis, suicide, suicide prevention, systematic review

## Abstract

**Background:**

Interventions at frequently used suicide locations that restrict access to means, encourage help-seeking, and increase the likelihood of intervention by a third party are effective in preventing suicide at such sites. However, there have been concerns that such efforts may displace suicides to other sites. It is important to synthesize the evidence on suicide displacement effects.

**Methods:**

We conducted a systematic search of Medline, PsycINFO, Scopus, and Google for eligible studies from their inception to February 20, 2025. Meta-analyses were conducted to assess the pooled effects of interventions on suicides at frequently used locations and other sites, and on overall suicides involving the same method.

**Results:**

Our search identified 17 studies. Meta-analyses showed a reduction in suicides at the intervention sites (pooled incidence rate ratio [IRR] 0.09, 95% confidence interval [95% CI] 0.04–0.21) and no evidence of changes in suicides at other sites after restricting access to means was deployed alone. The pooled IRR for nearby sites (same type) was 0.99 (95% CI 0.72–1.38); for other sites (same type), it was 0.99 (95% CI 0.76–1.29); and for other sites (different/unspecified type), it was 1.19 (95% CI 0.90–1.58). There was an overall reduction in suicides involving the same method during the post-intervention period (IRR 0.77, 95% CI 0.65–0.92). Similar patterns were observed when restricting access to means was assessed alone or with other interventions.

**Conclusions:**

Suicide numbers at other sites did not change after interventions such as restricting access to means were deployed at frequently used locations.

## Introduction

Suicide is a serious public health problem. Globally, it is estimated that approximately 720,000 lives are lost to suicide each year, equating to about 2,000 each day (World Health Organization, 2024). Certain sites have been identified as frequently used locations for suicides. These sites are typically specific, accessible, and public locations with a high incidence of suicides (Pirkis et al., 2015). These sites are also often iconic city structures, widely known by the public (Pirkis et al., 2015). Their frequent use for suicide may be influenced by contagion effects, where individuals are drawn to these sites after learning through media reports or word-of-mouth that other people have gone there to attempt suicide (Beautrais, 2007).

A recent Australian study (the first of its kind globally) found that suicides at frequently used locations accounted for 1% of all suicides and about 5% of suicides occurring in public places (Too et al., 2024). While these incidents are relatively uncommon, the public often becomes aware of them which can contribute to further suicides (Beautrais, 2007). Preventing these suicides is critical, and several interventions, such as restricting access to means (e.g., installing physical barriers), encouraging help-seeking (e.g., installing helpline signs), and increasing the likelihood of intervention by a third party (e.g., implementing police patrols), have proven effective in reducing suicide at these locations (Pirkis et al., 2013, 2015).

Despite there being good evidence supporting the effectiveness of various interventions in preventing suicide at frequently used locations (Pirkis et al., 2013, 2015), concerns have been raised about the potential for a displacement effect, where suicides shift to other sites following the introduction of these interventions (particularly for interventions that restrict access to means). To investigate this concern, several studies have examined the impact of interventions at frequently used locations on suicides at other sites. A previous meta-analysis from our group of the effectiveness of means restriction at jumping locations on suicides at the sites themselves also examined suicides at other sites. That meta-analysis pooled the data from six studies and found an increase in jumping suicides at the other sites, although there was an overall reduction in jumping suicides (Pirkis et al., 2013). The present systematic review and meta-analysis aimed to synthesize the findings from more studies to draw an updated and more comprehensive conclusion about this important research area. It considered displacement locations based on their distance from the intervention site and whether their site type was the same as or different from the intervention site. Our key question was: do interventions at frequently used suicide locations influence the incidence of suicide at other sites?

## Methods

### Study design

This systematic review and meta-analysis followed the Preferred Reporting Items for Systematic Reviews and Meta-Analyses (PRISMA) reporting guideline (Page et al., 2021).

### Search strategy

We searched three databases: MEDLINE, PsycINFO, and Scopus from their inception to February 20, 2025. We modified the search terms used in our previous meta-analyses of the effectiveness of interventions to reduce suicides at frequently used locations (Pirkis et al., 2013, 2015) to capture all studies included in these meta-analyses, given some of the included studies were identified through other sources. These search terms were reviewed by authors who were the experts in the field and mapped onto article titles: (suicid*) AND (hotspot* OR location* OR site* OR cliff OR lookout OR bridge OR building OR high-rise OR multi-storey OR viaduct OR rail OR railway OR metro OR subway OR woods OR forest OR skyscraper OR flyover* OR overpass OR ‘car park’ OR underground OR tube OR crossing OR road OR motorway OR highway OR reservoir OR coast OR jump* OR leap* OR fall OR height OR lie OR lying OR ‘carbon monoxide’ OR ‘car exhaust’ OR hang* OR firearm OR gun* OR burn* OR drown* OR fenc* OR barrier* OR parapet OR net* OR pit* OR sign* OR poster* OR helpline* OR surveillance* OR CCTV OR patrol*) (see full search strategy in eTable 1 in Supplement 1). We also searched the reference lists of included studies and key relevant reviews. Additionally, we searched grey literature from Google Advanced to find eligible studies using the following words: suicide site, suicide location, suicide hotspot, suicide jump, suicide bridge, suicide cliff, OR suicide railway.

### Selection criteria

We included studies published as original articles in scientific journals and reported in English. We also included unpublished studies in government documents, research reports, conference proceedings, pre- and post-print articles, and theses/dissertations. Eligible studies had to meet the following criteria framed using the Population, Intervention, Comparison, Outcome, and Study (PICOS) design tool (Centre for Reviews and Dissemination, 2009): used data from the general population (P); assessed intervention(s) at a frequently used suicide location (I); analyzed suicide deaths at other sites (C); measured suicide deaths at a frequently used suicide location as an outcome (O); and conducted a quasi-experiment (non-randomized studies with before-and-after designs) (S). Quasi-experimental studies were selected because it is practically and ethically challenging to conduct randomized controlled trial in the area. The interventions assessed in the study included restricting access to means, encouraging help-seeking, and/or increasing the likelihood of third-party intervention. These interventions could be implemented in isolation or in combination with each other. Studies were excluded if the intervention site was not a public location, such as inpatient psychiatric hospital wards.

### Data collection and extraction

One author (LST) performed the searches through the three databases and imported the records into Covidence. Two authors (SS and LST) conducted the title and abstract screening, assessed the full-text articles for eligibility, and extracted information from each included study. The extracted information was then cross-checked by another author (YT). Any inconsistencies in study eligibility and extracted information were discussed among SS, YT, and LST and resolved by consensus. The level of agreement between two authors in eligibility screening, eligibility assessment, and data extraction was extremely high (Cohen’s kappa [K] = 0.95, 95% CI = 0.91–0.99; K = 0.96, 95% CI = 0.93–1.00; and K = 0.97, 95% CI = 0.90–1.00, respectively). Contact was made with the primary authors of included articles to obtain extra/missing data for our meta-analyses and one provided data (Law & Yip, 2011). Two authors (YT and LST) searched and screened grey literature.

We grouped the included studies into three types of displacement locations. They were categorized based on their distance from the intervention site and/or whether their site type was the same as or different from the intervention site. These included (i) nearby sites of the same type, (ii) other sites of the same type, and (iii) other sites of different/unspecified type. To be classified as a nearby site, the displacement site must be located within 10 km of the intervention site or described as being in close proximity to it. Displacement sites that did not meet this criterion were classified as other sites. For site type, displacement locations with the same site type (e.g., bridge) as the intervention site (e.g., bridge) were classified as ‘same site type’, while those with a different site type (e.g., non-bridge locations) were classified as ‘different site type’. Locations without a specified site type (e.g., other jumping sites) were classified as ‘unspecified site type’.

We extracted information about the author(s) and publication year; the frequently used suicide location (intervention site); the suicide method typically used at the location; intervention(s) at the location; suicides at other sites; suicides involving the same method as that used at the intervention site; suicides by other methods; and overall suicides (i.e., sum of suicides involving the same method and suicides by other methods). We also extracted data on the duration of the observation period and suicide numbers in each category for the pre- and post-intervention periods.

### Risk of bias assessment

We used Risk of Bias in Non-randomized Studies of Interventions (ROBINS-I) (Sterne et al., 2016) to assess the quality of the included studies. The tool comprised seven bias domains and the assessors were required to judge the risk of bias of a study for each domain and then the overall risk based on the risk outcomes for these domains. Two authors (YT and LST) conducted the quality assessment (*K* = 0.88, 95% CI = 0.72–1.00), resolving any inconsistencies by consensus.

### Synthesis methods

We estimated the pooled IRR and 95% CI using a random-effects conditional model with an exact likelihood function. A random-effects model was selected over a fixed-effects model as study heterogeneity is common in meta-analysis, and the I^2^ for our models showed the presence of heterogeneity. This model groups observations within studies and accounts for between-study differences to estimate the population-averaged change in the suicide incidence from the pre-intervention period to the post-intervention period. An offset term for exposure time was included in the model. The pooled IRR indicates the change in the expected number of suicides per year at intervention sites, other sites, and suicides involving the same method after deploying interventions at the frequently used locations. If the IRR is 1, it suggests no change in suicides at the frequently used site or displacement sites following the deployment of interventions. If the IRR is less than 1, it means the interventions have reduced suicides at the site or that the number of suicides has declined at other locations. If the IRR is greater than 1, it suggests that the interventions have increased suicides at the site or that there is a rise in the number of suicides at other sites. If the 95% CI crosses 1, it indicates that the intervention or displacement effect is not statistically significant.

We ran six models to estimate the suicide displacement effects, with two models for each type of displacement location (one for studies with an intervention deployed alone, and another for studies with an intervention deployed with or without other interventions). We did the same to estimate suicides at intervention sites and suicides involving the same method. Heterogeneity between studies was assessed using the *I* ^2^ statistic and meta regression, and publication bias was assessed using funnel plots and Egger’s test.

If our results showed suicides did not displace to other sites during the post-intervention period, we conducted sensitivity analyses for suicides by other methods and overall suicides to examine if there was method substitution (i.e., change to another suicide method that was different from that typically used at the intervention site). All analyses were conducted using the ‘metafor’ package in the R program (version 4.4.0).

## Results

### Study selection

**Figure 1.**
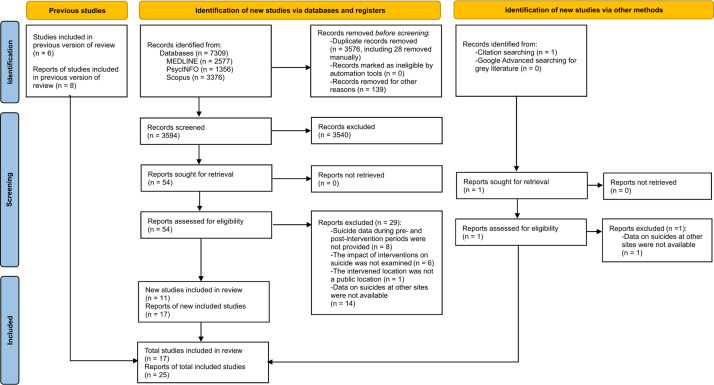
Flow diagram of included and non-included studies.

The initial search yielded 7,309 articles and one additional article from the citation search. After removing 3,576 duplicates and 139 non-original articles and screening the abstracts of 3,594 articles, 55 full-text articles were assessed for their eligibility. Thirty articles were excluded for the following reasons: (i) did not provide suicide data for the pre- and post-intervention periods (*n* = 8); (ii) did not examine the impact of interventions on suicide deaths (*n* = 6); (iii) did not examine a public location (*n* = 1); and (iv) did not provide data on suicides at other sites (*n* = 15). We considered articles about the same site as being the same study and included data from the article with more comprehensive information (e.g., a longer observation period; providing required data on other sites). As a result, 25 articles representing 17 studies of distinct sites met all inclusion criteria. Our search using Google Advanced did not yield any studies that met our inclusion criteria. Please refer to Figure 1 for the PRISMA flow diagram and eTable 2 in Supplement 1 for the PRISMA checklist.

### Overview of included studies

Table 1 shows the study descriptions of 17 included studies. All were conducted in high-income countries (i.e., one in Austria, one in Hong Kong, one in Sweden, one in Switzerland, two in Canada, two in New Zealand, two in the USA, two in the UK, and five in Australia). Of these studies, 13 focused on suicide by jumping from a height (11 bridges and two cliffs), three on suicide by jumping in front of a train, and one on suicide by carbon monoxide poisoning. Sixteen studies assessed the impact of restricting access to means alone (*n* = 14) or paired with interventions aimed at encouraging help-seeking and increasing the likelihood of intervention by a third party (*n* = 2). Only one study examined an intervention that encouraged help-seeking alone (King & Frost, 2005). Therefore, we were not able to estimate the pooled effect of this intervention alone and excluded this study in our subsequent meta-analyses. We found no studies that assessed increasing the likelihood of intervention by a third party as a standalone intervention.

**Table 1.** Descriptions of included studies

	Author(s) and date	Frequently used suicide location (intervention site)	Suicide method typically used at intervention site	Interventions	Other sites		Suicides involving the same method as that used at the intervention site	Suicides by other methods	All suicides
					Same site type	Different/Unspecified site type			
1	Beautrais (2001)	‘Bridge A’^a^ in ‘City Z’^a^, New Zealand	Jumping from height	Removal of safety barriers (reversal design)	N/A	Other jumping sites in ‘City Z’^a^	Suicides by jumping ‘City Z’^a^	N/A	N/A
2	Bennewith, Nowers, and Gunnell (2007)^c^; Bennewith, Nowers, and Gunnell (2011)	Clifton Suspension Bridge, Bristol, UK	Jumping from height	Two meters high wire barriers were installed on the main span of the bridge in December 1998	N/A	Other jumping sites in Bristol, UK	Suicides by jumping from height in Bristol, UK	N/A	N/A
3	Clapperton et al. (2022)	41 railway sites where level crossings were removed, Victoria, Australia	Jumping in front of a train	Level-crossings of 41 sites were removed at different time points (dates were not specified in the article). This resulted in railway tracks went under/over the road, making access to the tracks difficult.	41 sites with level-crossings, Victoria, Australia	N/A	N/A	N/A	N/A
4	Deisenhammer and Pitschieler (2024)	Europe Bridge, Tyrol, Austria	Jumping from height	Solid but not insurmountable barriers were erected on the bridge in 2011	Other bridges in the proximity of Europe Bridge (Innsbruck City and Innsbruck County) Other bridges in other counties in Tyrol	Natural sites in Tyrol	Suicides by jumping from height in Tyrol	N/A	N/A
5	Dwyer et al. (2023)	West Gate Bridge, Victoria, Australia	Jumping from height	Installation of a safety barrier between March 2009 and March 2011	Other bridges, Victoria, Australia	Non-bridge locations for jumping in Victoria, Australia	Suicides by jumping from height in Victoria, Australia	Suicides by means other than jumping from height in Victoria	All suicides in Victoria
6	Fredin-Knutzén, Hadlaczky, Andersson, and Sokolowski (2022)	High-speed tracks at Valby railway station, suburb of Stockholm, Sweden	Jumping in front of a train on high-speed tracks	Installation of 1-m-high mid-track fences partially restricting access to high-speed train tracks between during/end of 2013 and the beginning of 2014	High-speed tracks at six nearby railway stations on the same line without mid-track fencing/other separator in Stockholm, Sweden	N/A	N/A	N/A	N/A
7	King and Frost (2005)	26 signed car parks, New Forest, Hampshire, UK	Carbon monoxide poisoning (by car exhaust in isolated car parks)	Installation of signs displaying Samaritans’ national telephone number in 26 carparks in 1998	114 unsigned car parks, New Forest Hampshire, UK	Elsewhere in New Forest	Suicides by carbon monoxide poisoning in New Forest	Suicides by other methods in New Forest	All suicides in New Forest
8	Kõlves Leske, and De Leo (2023)	Story Bridge, Brisbane, Australia	Jumping from height	Installation of physical barrier completed in December 2015 Lifeline phones were installed in 2012 Surveillance cameras were installed in 2012	Other bridges and cliffs in Brisbane	Man-made structures in Inner Brisbane	N/A	N/A	N/A
9	Law et al. (2009); Law and Yip (2011)^b^	Underground railway system, Hong Kong	Jumping in front of a train	Installation of platform screen doors on 71 platforms in 30 underground stations on three prominent transit lines; work began in 2002, and ended in 2005, but most of the busiest station platforms were sealed in the first year	Underground railway stations without platform screen doors on the same line	N/A	Underground railway suicides in Hong Kong	Suicides by means other than jumping in front of a train in Hong Kong	All suicides in Hong Kong
10	Law, Sveticic, and De Leo (2014)	Gateway Bridge, Brisbane, Australia	Jumping from height	Installation of 3.3-m-high safety barriers in 1993; replaced with 3.6 m safety barriers in November 2010	Story Bridge (8.9 km from Gateway Bridge) Other bridges in Brisbane, Australia	Non-bridge jumping sites in Brisbane	Suicides by jumping from height in Brisbane	Suicides by means other than jumping from height in Brisbane	All suicides in Brisbane
11	Lockley et al. (2014); Ross, Koo, and Kolves (2020); Torok et al. (2023)^c^	Gap Park, Sydney, Australia	Jumping from height	Installation of 1.3-m-high inwardly-curved fencing at two high-risk points in July 2011 Installation of crisis telephones and signages displaying a dedicated lifeline telephone number in February 2010. Additional signages installed in July 2011 Installation of CCTV cameras in February 2010	North Head (9.1 km from Gap Park)	Jumping sites in the Sydney ‘local’ areas defined by the authors	Suicides by jumping from height in the Sydney ‘broader’ areas defined by the authors	N/A	N/A
12	Lester (1993); O’Carroll, Silverman, and Berman (1994); Berman, Athey, and Nestadt (2022)^c^	Ellington Bridge, Washington DC, USA	Jumping from height	Installation of 2.4-m-high fence in late January 1986	Taft Bridge (483 m from Ellington Bridge) Other bridges in Washington	N/A	N/A	N/A	All suicides in Washington
13	Pelletier (2007)	Memorial Bridge, Augusta, Maine, USA	Jumping from height	Installation of 3.4-m-high fence on either side bridge in 1983	Other bridges in Augusta	Other jumping sites in Augusta	Suicides by jumping from height in Augusta	N/A	N/A
14	Perron et al. (2013)	Jacques-Cartier Bridge, Montreal, Quebec, Canada	Jumping from height	Extension of existing 1.1 m fence by a further 1.4 m with inwardly curving top in 2004	Other bridges on Montréal Island and Montérégie, Quebec, Canada	Non-bridge jumping sites on Montréal Island and Montérégie, Quebec, Canada	Suicides by jumping from height on Montréal Island and Montérégie, Quebec, Canada (except unknown bridges connecting Montréal Island and Montérégie)	Suicides by means other than jumping from height (but including jumping from unknown bridges connecting Montréal Island and Montérégie)	All suicides in Montréal Island and Montérégie
15	Reisch and Michel (2005)	Muenster Terrace, Bern, Switzerland	Jumping from height	Installation of a 4-m-wide metal mesh net, 7 m below the top of terrace in 1998	Kirchenfeld Bridge	N/A	Suicides by jumping in Bern	N/A	N/A
16	Sinyor and Levitt (2010); Sinyor et al. (2017)^c^; Sinyor et al. (2024)	Bloor Street Viaduct, Toronto, Ontario, Canada	Jumping from height	Construction of 5-m-high barrier between April 2002 and June 2003	Nearest bridge of similar size in Toronto Other walking distance bridges in Toronto (within 5 km from the intervention site)	Building sites in Toronto	Suicides by jumping from height in Toronto	Suicides by means other than jumping from height in Toronto	All suicides in Toronto
17	Skegg and Herbison (2009)	Lawyers Head Cliff, Dunedin, New Zealand	Jumping from height	Road access blocked in 2006 due to maintenance	Other cliffs	N/A	Suicides by jumping from height in the Dunedin City Police district	Suicides by means other than jumping from height in the Dunedin City Police district	All suicides in the Dunedin City Police district

*Note*: N/A, not available.

a This city was anonymized in the article.

b Data for this study were provided by the authors upon request.

c The data from this article were selected and included in our meta-analysis because it included more comprehensive information (e.g., a longer observation period, providing required data on other sites).

Of the 16 included studies assessing the impact of restricting access to means with or without other interventions, 6 provided data on a nearby site (same type), 12 on other sites (same type), and 10 on other sites (different/unspecified type).

### Risk of bias in studies

Of the 16 studies, 14 were considered as having a moderate risk of bias (up to two domains with moderate bias) and two as having a low risk (all domains with low bias). The studies with a moderate risk commonly did not consider the time taken to deploy interventions (which may influence the effect of interventions on suicides at other sites) and/or did not adjust for time trends and/or population size in their analysis. Please see eTable 3 in Supplement 1 for the details on the risk of bias assessment.

### Pooled estimates of incidence rate ratios at intervention sites

**Figure 2.**
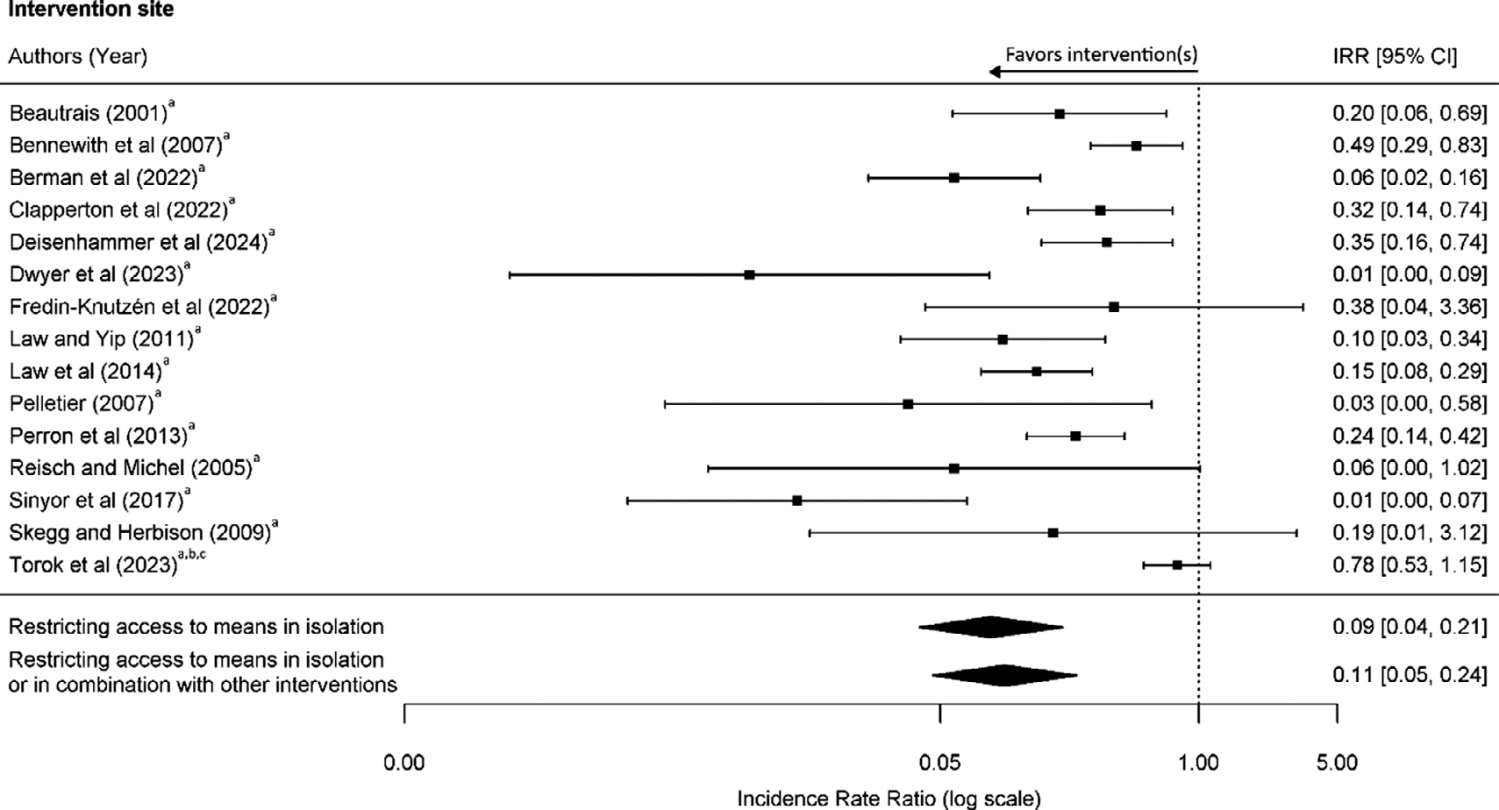
Risk of suicide at frequently used location following interventions at the location. Interventions: ^a^restricting access to means, ^b^encouraging help-seeking, and ^c^increasing the likelihood of intervention by a third party.

We found a total of 645 suicides occurring over 131.5 study-years at intervention sites during pre-intervention periods (an unweighted mean of 78.2 suicides per year) and 129 suicides occurring over 145 study-years during post-intervention periods (an unweighted mean of 18.6 suicides per year) (eTable 4 in Supplement 1). The pooled IRR was 0.09 (95% CI 0.04–0.21) for restricting access to means delivered alone and 0.11 (95% CI 0.05–0.24) for restricting access to means delivered alone or with other interventions (Figure 2). These findings suggest that restricting access to means was highly effective, with the number of suicides at the intervention sites reduced by 91% and 89%, respectively.

### Pooled estimates of incidence rate ratios at other sites

**Figure 3.**
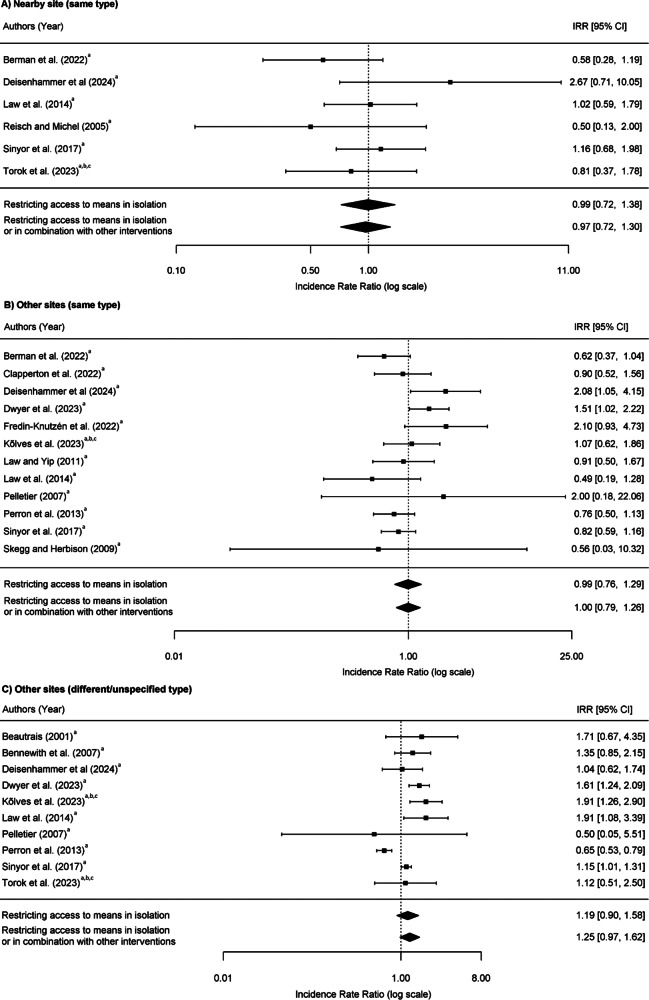
Risk of suicide at other sites following interventions at frequently used suicide location. A) Nearby site (same type); B) Other sites (same type); and C) Other sites (different/unspecified type). Interventions: ^a^restricting access to means, ^b^encouraging help-seeking, and ^c^increasing the likelihood of intervention by a third party.

As shown in eTable 5 in supplement 1, for nearby sites (same site type), a total of 71 suicides occurred over 41 study-years during pre-intervention periods (an unweighted mean of 11.6 suicides per year) and 155 suicides occurred over 82 study-years during post-intervention periods (an unweighted mean of 10.6 suicides per year). The pooled IRR was 0.99 (95% CI 0.72–1.38) for means restriction delivered in isolation and 0.97 (95% CI 0.72–1.30) for means restriction delivered in isolation or in combination with other interventions (Figure 3a). This suggests that there was no change in suicides at nearby sites after interventions were deployed at the site of concern.

For other sites (same type), 397 suicides took place over 127.5 study-years during pre-intervention periods (an unweighted mean of 39.5 suicides per year) and 321 suicides occurred over 130 study-years during post-intervention periods (an unweighted mean of 37.9 suicides per year). The pooled IRR was 0.99 (95% CI 0.76–1.29) for means restriction delivered alone, and 1.00 (95% CI 0.79–1.26) for means restriction delivered with or without other interventions (Figure 3b). This indicates that suicides did not shift to other sites of the same type following the deployment of the intervention(s).

For other sites (different/unspecified type), 1,197 suicides occurred over 97.9 study-years during pre-intervention periods (an unweighted mean of 109.7 suicides per year) and 1,026 suicides occurred over 97.0 study-years during post-intervention periods (an unweighted mean of 118.9 suicides per year). The pooled IRR was 1.19 (95% CI 0.90–1.58) for means restriction delivered alone, and 1.25 (95% CI 0.97–1.62) means restriction delivered alone or with other interventions (Figure 3c). This suggests a slight increase in suicides at other sites of a different or unspecified type following the deployment of an intervention that restricted access to means, either alone or in combination with other interventions. However, the effect ranged from a small reduction in suicides to a moderate increase, indicating that the evidence for a clear, consistent displacement effect to these locations is weak.

### Pooled estimates of incidence rate ratios for suicides involving the same method

**Figure 4.**
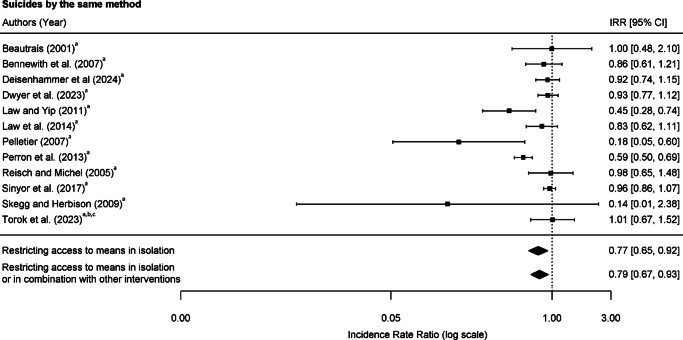
Risk of suicide by the same suicide method following interventions at frequently used suicide location. Interventions: ^a^restricting access to means, ^b^encouraging help-seeking, and ^c^increasing the likelihood of intervention by a third party.

In terms of suicides involving the same suicide method as that used at the intervention sites, 2,141 suicides were found over 101.9 study-years during the pre-intervention periods (an unweighted mean of 224.9 suicides per year) and 1,573 suicides over 102.0 study-years during the post-intervention periods (an unweighted mean of 182.7 suicides per year). The pooled IRR was 0.77 (95% CI 0.65–0.92) for means restriction delivered alone and 0.79 (95% CI 0.67–0.93) for means restriction delivered with or without other interventions (Figure 4 and eTable 6 in Supplement 1), suggesting a decrease in suicides involving the same method in both approaches.

## Pooled estimates of incidence rate ratios for suicides by other methods and all suicides

Our sensitivity analyses included six studies that examined suicides by other methods for means restriction delivered alone found the pooled IRR was 1.04 (95% CI 0.90–1.22) (eFigure 1 in Supplement 1). For all suicides, the pooled IRR was 0.97 (95% CI 0.83–1.14) for means restriction delivered alone (eFigure 2 in Supplement 1). These results suggest no change in suicides by other methods, nor in the overall suicides.

### Between-study heterogeneity and publication bias

The I^2^ statistics indicated varying degrees of heterogeneity among studies across 12 models. For means restriction in isolation, the I^2^ was 86.3% for intervention sites, 0% for nearby sites (same type), 49.7% for other sites (same type), 80% for other sites (different/unspecified type), and 72.2% for suicides involving the same suicide method. Similar patterns were found for means restriction with or without other interventions. The 0% I^2^ for nearby sites may suggest bias due to the small number of studies included (von Hippel, 2015).

Meta-regression analysis revealed that the high heterogeneity observed in the model for intervention sites was primarily explained by study country (22.7%). For other sites (different/unspecified type), the high heterogeneity was mainly explained by the duration of the pre-intervention period (29.9%), the duration of the post-intervention period (11.8%), and the study country (6.9%). The high heterogeneity for suicides involving the same suicide method was mainly explained by study country (11.4%), suicide method (9.5%), and type of suicide location (6.5%). Study country, suicide method, type of suicide location, and both the pre- and post-intervention durations were not significant moderators in most models, except for the pre-intervention duration in the model for other sites (different/unspecified type), which was significant (*p* = 0.002).

The Egger’s test for funnel plot asymmetry was non-significant for all models, except for the model of intervention sites (*p* = 0.02) (eFigures 3 and 4 in Supplement 1). The significant value from the Egger’s test for intervention sites became non-significant after we considered population size in the meta-analysis (including four studies) and the positive intervention effects remained (IRR = 0.18, 95% CI 0.12–0.27).

## Discussion

This systematic review and meta-analysis examined changes in suicide deaths following efforts to restrict access to means at frequently used sites for suicide with or without other concurrent interventions. We found a reduction in suicides at such sites, no evidence of changes in suicides at other sites, and a reduction in suicides involving the same method as used at the intervention sites.

Our findings showing no evidence of suicide displacement effects represent an advance because they contradict the displacement argument, which is often used to oppose the installation of structural interventions at sites of concern, and suggest that these interventions at such sites can truly make a difference in preventing suicides. Our findings showing a decrease in suicides involving the same suicide method are likely to be driven by the effectiveness of interventions at sites in reducing suicides at these locations.

Although our pooled results indicated non-significant changes in suicides at other sites, our forest plots showed an increase in suicides at these sites in some individual studies. However, it is crucial to note that several individual studies reported non-significant results after adjusting for population size and/or time trends (Dwyer et al., 2023; Law, Sveticic, & De Leo, 2014; Sinyor et al., 2017). We could not adjust for these factors in our meta-analysis because this information was not consistently available in the included studies. This phenomenon highlights the importance for future research to control for confounding to provide more accurate results. It also explains why our present findings are different from those in our previous meta-analysis that found an increase of suicides at other sites after restricting access to means was deployed at sites (Pirkis et al., 2013). The previous finding was predominantly driven by an older study in Toronto, Canada (Sinyor & Levitt, 2010), which the authors later updated and, after including a longer post-intervention period that included both high and low levels of media coverage on the intervention site, found no change in suicides at other sites (Sinyor et al., 2017).

Our non-significant findings for other sites may be explained by the possibility that these sites were not as well-known as the frequently used locations where interventions were deployed. At-risk individuals may select frequently used locations because they are aware that others have previously gone to these sites to end their lives. Yet, they may be unaware of the availability and accessibility of the other sites for suicide. As a result, they do not attempt suicide at those sites when the intervention site becomes less accessible. Another possible explanation for these findings is that the frequently used location may hold a particular meaning for at-risk individuals, making them less likely to choose alternative sites after it becomes less accessible (Lam, Kinney, & Bell, 2017). Both explanations are supported by our findings from a previous Australian study that over 70% of individuals who died by suicide at frequently used locations (e.g., cliffs/bridges) lived more than 5 km from their chosen suicide location (Too et al., 2019). This suggests that individuals may select a site based on reputation or personal meaning which makes them prepared to travel a distance to the site.

Our sensitivity analysis found no change in suicides by other methods, suggesting that a decrease in suicides by one method does not increase suicides by other methods. We also found no change in all suicides. This could be due to the suicide method used at the intervention site is not that frequent (e.g., ~5% [Dwyer et al., 2023; Law, Sveticic, & De Leo, 2014]); therefore, the reduction in suicides involving the same method may not have had a discernible impact on overall suicide numbers (Dwyer et al., 2023; Law, Sveticic, & De Leo, 2014).

## Limitations

Our study should be interpreted with the following limitations. First, we might have missed some studies in our searches, such as those that were not published in scientific journals. Second, this review was not pre-registered in advance with PROSPERO or a similar registry. This means some risk of selection bias might exist. However, outcome selection bias should be minimal because we included all primary outcomes considered in the area. Third, we had limited data on suicides for nearby sites, which limits our conclusion for these sites. Fourth, potential publication bias was detected from the Egger’s test for intervention sites, and this bias changed to non-significant after adjusting for population size in the model. This suggests that population size should be considered in this evaluation research to avoid overestimating the intervention effect. Fifth, we observed a possible, though uncertain, increase in suicides at other sites of a different or unspecified type. Further research is needed to confirm the displacement effect at these sites. This research should consider population size in its evaluation as population size increases over time. Without this consideration, the displacement effect is likely to be overestimated. Lastly, we could not assess the impact of increasing the likelihood of third-party interventions in isolation on suicide at other sites as this has not been studied. Future study should examine this intervention alone because, like restricting access to means, patrolling by third party also makes selected sites less accessible and may result in suicide displacement to other sites.

## Conclusions

Our findings are critical in suicide prevention, suggesting that interventions that restrict access to means at sites with or without other interventions can prevent suicide and that the suicide displacement argument is likely to be false and not a valid reason to oppose the deployment of such interventions. Nevertheless, it is still important to be vigilant for potential site displacement, particularly if nearby sites have also been used for suicide, in which case interventions should also be introduced.

## Supporting information

Too et al. supplementary material Too et al. supplementary material

## Data Availability

Data used in this study are presented in eTable 4, eTable 5, and eTable 6.

## Supplementary material

The supplementary material for this article can be found a http://doi.org/10.1017/S0033291725100792.
