# Supplementary material for: Impact of interventions at frequently used suicide locations on occurrence of suicides at other sites: a systematic review and meta-analysis
Source: Psychol Med. 2025 Jun 24;55:e168. doi: 10.1017/S0033291725100792 (PMC12201958; doi:10.1017/S0033291725100792)
Supplement: Too et al. supplementary material [file S0033291725100792sup001.docx]

**eTable 1: Search Strategy**

| **Order** | **Search Term** | **Results** |
| --- | --- | --- |
|  | **Database: Ovid MEDLINE** |  |
| 1 | (suicid* and (hotspot* or location* or site* or cliff or lookout or bridge or building or high-rise or multi-storey or viaduct or rail or railway or metro or subway or woods or forest or skyscraper or flyover* or overpass or "car park" or underground or tube or crossing or road or motorway or highway or reservoir or coast or jump* or leap* or fall or height or lie or lying or "carbon monoxide" or "car exhaust" or hang* or firearm or gun* or burn* or drown* or fenc* or barrier* or parapet or net* or pit* or sign* or poster* or helpline* or surveillance* or CCTV or patrol*)).ti. | 2830 |
| 2 | limit 1 to english language | 2577 |
|  |  |  |
|  | **Database: Ovid PsycINFO** |  |
| 1 | (suicid* and (hotspot* or location* or site* or cliff or lookout or bridge or building or high-rise or multi-storey or viaduct or rail or railway or metro or subway or woods or forest or skyscraper or flyover* or overpass or "car park" or underground or tube or crossing or road or motorway or highway or reservoir or coast or jump* or leap* or fall or height or lie or lying or "carbon monoxide" or "car exhaust" or hang* or firearm or gun* or burn* or drown* or fenc* or barrier* or parapet or net* or pit* or sign* or poster* or helpline* or surveillance* or CCTV or patrol*)).ti. | 1478 |
| 2 | limit 1 to english language | 1356 |
|  |  |  |
|  | **SCOPUS** |  |
| 1 | (TITLE(suicid*) AND TITLE(hotspot* OR location* OR site* OR cliff OR lookout OR bridge OR building OR high-rise OR multi-storey OR viaduct OR rail OR railway OR metro OR subway OR woods OR forest OR skyscraper OR flyover* OR overpass OR "car park" OR underground OR tube OR crossing OR road OR motorway OR highway OR reservoir OR coast OR jump* OR leap* OR fall OR height OR lie OR lying OR "carbon monoxide" OR "car exhaust" OR hang* OR firearm OR gun* OR burn* OR drown* OR fenc* OR barrier* OR parapet OR net* OR pit* OR sign* OR poster* OR helpline* OR surveillance* OR CCTV OR patrol*)) AND ( LIMIT-TO ( LANGUAGE,"English" ) ) | 3376 |
|  |  |  |
|  | **Google Advanced** |  |
| 1 | Find pages with all these words: suicide site, suicide location, suicide hotspot, suicide jump, suicide bridge, suicide cliff, OR suicide railway | All pages screened |

**eTable 2. PRISMA 2020 checklist**

| **Section and Topic** | **Item #** | **Checklist item** | **Location where item is reported** |
| --- | --- | --- | --- |
| **TITLE** | | |  |
| Title | 1 | Identify the report as a systematic review. | Title page |
| **ABSTRACT** | | |  |
| Abstract | 2 | See the PRISMA 2020 for Abstracts checklist. | Page 3 |
| **INTRODUCTION** | | |  |
| Rationale | 3 | Describe the rationale for the review in the context of existing knowledge. | Page 4-5 |
| Objectives | 4 | Provide an explicit statement of the objective(s) or question(s) the review addresses. | Page 5 |
| **METHODS** | | |  |
| Eligibility criteria | 5 | Specify the inclusion and exclusion criteria for the review and how studies were grouped for the syntheses. | Page 6-7 |
| Information sources | 6 | Specify all databases, registers, websites, organisations, reference lists and other sources searched or consulted to identify studies. Specify the date when each source was last searched or consulted. | Page 5-6 |
| Search strategy | 7 | Present the full search strategies for all databases, registers and websites, including any filters and limits used. | eTable 1 in Supplement 1 |
| Selection process | 8 | Specify the methods used to decide whether a study met the inclusion criteria of the review, including how many reviewers screened each record and each report retrieved, whether they worked independently, and if applicable, details of automation tools used in the process. | Page 6-8 |
| Data collection process | 9 | Specify the methods used to collect data from reports, including how many reviewers collected data from each report, whether they worked independently, any processes for obtaining or confirming data from study investigators, and if applicable, details of automation tools used in the process. | Page 7 |
| Data items | 10a | List and define all outcomes for which data were sought. Specify whether all results that were compatible with each outcome domain in each study were sought (e.g. for all measures, time points, analyses), and if not, the methods used to decide which results to collect. | Page 7 |
|  | 10b | List and define all other variables for which data were sought (e.g. participant and intervention characteristics, funding sources). Describe any assumptions made about any missing or unclear information. | Table 1 |
| Study risk of bias assessment | 11 | Specify the methods used to assess risk of bias in the included studies, including details of the tool(s) used, how many reviewers assessed each study and whether they worked independently, and if applicable, details of automation tools used in the process. | Page 8 |
| Effect measures | 12 | Specify for each outcome the effect measure(s) (e.g. risk ratio, mean difference) used in the synthesis or presentation of results. | Page 8-9 |
| Synthesis methods | 13a | Describe the processes used to decide which studies were eligible for each synthesis (e.g. tabulating the study intervention characteristics and comparing against the planned groups for each synthesis (item #5)). | Page 8-9 |
|  | 13b | Describe any methods required to prepare the data for presentation or synthesis, such as handling of missing summary statistics, or data conversions. | Page 8-9 |
|  | 13c | Describe any methods used to tabulate or visually display results of individual studies and syntheses. | Page 8-9 |
|  | 13d | Describe any methods used to synthesize results and provide a rationale for the choice(s). If meta-analysis was performed, describe the model(s), method(s) to identify the presence and extent of statistical heterogeneity, and software package(s) used. | Page 8-9 |
|  | 13e | Describe any methods used to explore possible causes of heterogeneity among study results (e.g. subgroup analysis, meta-regression). | Page 9 |
|  | 13f | Describe any sensitivity analyses conducted to assess robustness of the synthesized results. | Page 9 |
| Reporting bias assessment | 14 | Describe any methods used to assess risk of bias due to missing results in a synthesis (arising from reporting biases). | Page 8 |
| Certainty assessment | 15 | Describe any methods used to assess certainty (or confidence) in the body of evidence for an outcome. | Page 8-9 |
| **RESULTS** | | |  |
| Study selection | 16a | Describe the results of the search and selection process, from the number of records identified in the search to the number of studies included in the review, ideally using a flow diagram. | Page 9-10, Figure 1 |
|  | 16b | Cite studies that might appear to meet the inclusion criteria, but which were excluded, and explain why they were excluded. | Page 9-10 |
| Study characteristics | 17 | Cite each included study and present its characteristics. | Page 10-11, Table 1 |
| Risk of bias in studies | 18 | Present assessments of risk of bias for each included study. | eTable 3 in Supplement 1 |
| Results of individual studies | 19 | For all outcomes, present, for each study: (a) summary statistics for each group (where appropriate) and (b) an effect estimate and its precision (e.g. confidence/credible interval), ideally using structured tables or plots. | Page 11-13 |
| Results of syntheses | 20a | For each synthesis, briefly summarise the characteristics and risk of bias among contributing studies. | Page 11 |
|  | 20b | Present results of all statistical syntheses conducted. If meta-analysis was done, present for each the summary estimate and its precision (e.g. confidence/credible interval) and measures of statistical heterogeneity. If comparing groups, describe the direction of the effect. | Page 11-13 |
|  | 20c | Present results of all investigations of possible causes of heterogeneity among study results. | Page 13-14 |
|  | 20d | Present results of all sensitivity analyses conducted to assess the robustness of the synthesized results. | Page 13 |
| Reporting biases | 21 | Present assessments of risk of bias due to missing results (arising from reporting biases) for each synthesis assessed. | Page 13 |
| Certainty of evidence | 22 | Present assessments of certainty (or confidence) in the body of evidence for each outcome assessed. | Page 13 |
| **DISCUSSION** | | |  |
| Discussion | 23a | Provide a general interpretation of the results in the context of other evidence. | Page 14-16 |
|  | 23b | Discuss any limitations of the evidence included in the review. | Page 16-17 |
|  | 23c | Discuss any limitations of the review processes used. | Page 16-17 |
|  | 23d | Discuss implications of the results for practice, policy, and future research. | Page 17 |
| **OTHER INFORMATION** | | |  |
| Registration and protocol | 24a | Provide registration information for the review, including register name and registration number, or state that the review was not registered. | Page 16 |
|  | 24b | Indicate where the review protocol can be accessed, or state that a protocol was not prepared. | Page 16 |
|  | 24c | Describe and explain any amendments to information provided at registration or in the protocol. | Page 16 |
| Support | 25 | Describe sources of financial or non-financial support for the review, and the role of the funders or sponsors in the review. | Page 18 |
| Competing interests | 26 | Declare any competing interests of review authors. | Page 18 |
| Availability of data, code and other materials | 27 | Report which of the following are publicly available and where they can be found: template data collection forms; data extracted from included studies; data used for all analyses; analytic code; any other materials used in the review. | Page 19 |

**eTable 3: Details of risk of bias assessment**

| **Author(s) and date** | **Domain 1** | **Domain 2** | **Domain 3** | **Domain 4** | **Domain 5** | **Domain 6** | **Domain 7** | **Overall bias** |
| --- | --- | --- | --- | --- | --- | --- | --- | --- |
|  | **Bias due to confounding** | **Bias in selection of participants into the study** | **Bias in classification of interventions** | **Bias due to deviations from intended interventions** | **Bias due to missing data** | **Bias in measurement of outcomes** | **Bias in selection of the reported result** |  |
| Beautrais (2001) | Moderate risk: analysis was not controlled for potential confounders. | Low risk: the study used suicide data and did not involve participant selection. | Low risk: intervention status was well defined. | Low risk: no deviations from intended intervention. | Low risk: it is common that suicide number is underreported due to misclassification to other deaths. | Low risk: the study excluded suicides occurred during the time when barriers were removed in the analysis. | Low risk: results on the intervention site and other sites were reported in the same way. | Moderate risk |
| Bennewith et al. (2007) | Moderate risk: analysis was not controlled for potential confounders | Low risk: the study used suicide data and did not involve participant selection. | Low risk: intervention status was well defined. | Low risk: no deviations from intended intervention. | Low risk: it is common that suicide number is underreported due to misclassification to other deaths. | Moderate risk: suicides occurred during barrier installation were not excluded in the analysis. | Low risk: results on the intervention site and other sites were reported in the same way. | Moderate risk |
| Berman et al. (2022) | Moderate risk: analysis was not controlled for potential confounders. | Low risk: the study used suicide data and did not involve participant selection. | Low risk: intervention status was well defined. | Low risk: no deviations from intended intervention. | Low risk: it is common that suicide number is underreported due to misclassification to other deaths. | Low risk: suicides occurred during fencing construction were excluded in the analysis | Low risk: results on the intervention site and other sites were reported in the same way. | Moderate risk |
| Clapperton et al. (2022) | Moderate risk: analysis was not controlled for population size. | Low risk: the study used suicide data and did not involve participant selection. | Low risk: intervention status was well defined. | Low risk: no deviations from intended intervention. | Low risk: it is common that suicide number is underreported due to misclassification to other deaths. | Moderate risk: the study did not mention whether the intervention sites (after level crossing removal) were accessible within 500 metres radius. | Low risk: results on the intervention site and other sites were reported in the same way. | Moderate risk |
| Deisenhammer et al. (2024) | Moderate risk: analysis was not controlled for potential confounders. | Low risk: the study used suicide data and did not involve participant selection. | Low risk: intervention status was well defined. | Low risk: no deviations from intended intervention. | Low risk: it is common that suicide number is underreported due to misclassification to other deaths. | Low risk: suicides occurred during barriers installation were excluded in the analysis | Low risk: results on the intervention site and other sites were reported in the same way. | Moderate risk |
| Dwyer et al. (2023) | Low risk: analysis was controlled for time and monthly population size. | Low risk: the study used suicide data and did not involve participant selection. | Low risk: intervention status was well defined. | Low risk: no deviations from intended intervention. | Low risk: it is common that suicide number is underreported due to misclassification to other deaths. | Low risk: suicide outcome was measured during pre-, during-, and post-intervention periods. | Low risk: results on the intervention site and other sites were reported in the same way. | Low risk |
| Fredin-Knutzén et al. (2022) | Moderate risk: analysis was not controlled for potential confounders | Low risk: the study used suicide data and did not involve participant selection. | Low risk: intervention status was well defined. | Low risk: no deviations from intended intervention. | Low risk: it is common that suicide number is underreported due to misclassification to other deaths. | Moderate risk: suicides occurred during fencing installation were not excluded in the analysis. | Low risk: results on the intervention site and other sites were reported in the same way. | Moderate risk |
| Kõlves et al. (2023) | Moderate risk: analysis was not controlled for potential confounders. | Low risk: the study used suicide data and did not involve participant selection. | Low risk: intervention status was well defined. | Low risk: no deviations from intended intervention. | Low risk: it is common that suicide number is underreported due to misclassification to other deaths. | Moderate risk: suicides occurred during barriers installation were not excluded in the analysis. | Low risk: results on the intervention site and other sites were reported in the same way. | Moderate risk |
| Law and Yip (2011) | Low risk: analysis was adjusted for time trend, gender, and population growth rate. | Low risk: the study used suicide data and did not involve participant selection. | Low risk: intervention status was well defined. | Low risk: no deviations from intended intervention. | Low risk: it is common that suicide number is underreported due to misclassification to other deaths. | Low risk: suicides occurred during the first year of platform screen doors installation was excluded in the analysis. | Low risk: results on the intervention site and other sites were reported in the same way. | Low risk |
| Law et al. (2014) | Low risk: suicide rates were used in the analysis. | Low risk: the study used suicide data and did not involve participant selection. | Low risk: intervention status was well defined. | Low risk: no deviations from intended intervention. | Low risk: it is common that suicide number is underreported due to misclassification to other deaths. | Moderate risk: suicides occurred during barriers installation were not excluded in the analysis. | Low risk: results on the intervention site and other sites were reported in the same way. | Moderate risk |
| Pelletier (2007) | Moderate risk: analysis was not controlled for potential confounders. | Low risk: the study used suicide data and did not involve participant selection. | Low risk: intervention status was well defined. | Low risk: no deviations from intended intervention. | Low risk: it is common that suicide number is underreported due to misclassification to other deaths. | Moderate risk: suicides occurred during fencing installation were not excluded in the analysis. | Low risk: results on the intervention site and other sites were reported in the same way. | Moderate risk |
| Perron et al. (2013) | Moderate risk: analysis was not controlled for potential confounders. | Low risk: the study used suicide data and did not involve participant selection. | Low risk: intervention status was well defined. | Low risk: no deviations from intended intervention. | Low risk: it is common that suicide number is underreported due to misclassification to other deaths. | Low risk: suicides occurred during barrier construction were excluded in the analysis. | Low risk: results on the intervention site and other sites were reported in the same way. | Moderate risk |
| Reisch and Michel (2005) | Moderate risk: analysis was not controlled for potential confounders. | Low risk: the study used suicide data and did not involve participant selection. | Low risk: intervention status was well defined. | Low risk: no deviations from intended intervention. | Low risk: it is common that suicide number is underreported due to misclassification to other deaths. | Moderate risk: suicides occurred during barrier installation were not excluded in the analysis. | Low risk: results on the intervention site and other sites were reported in the same way. | Moderate risk |
| Sinyor et al. (2017) | Low risk: analysis accounted for overdispersion and adjusted for time and population size. The analysis also included a longer post-intervention period that contained both high and low levels of media coverage on the intervention site. | Low risk: the study used suicide data and did not involve participant selection. | Low risk: intervention status was well defined. | Low risk: no deviations from intended intervention. | Low risk: it is common that suicide number is underreported due to misclassification to other deaths. | Moderate risk: suicides occurred during barrier installation were not excluded in the analysis. | Low risk: results on the intervention site and other sites were reported in the same way. | Moderate risk |
| Skegg and Herbison (2009) | Moderate risk: analysis was not controlled for potential confounders. | Low risk: the study used suicide data and did not involve participant selection. | Low risk: intervention status was well defined. | Low risk: no deviations from intended intervention. | Low risk: it is common that suicide number is underreported due to misclassification to other deaths. | Low risk: road closure was considered as intervention that restricts access to means. No barrier construction involved. | Low risk: results on the intervention site and other sites were reported in the same way. | Moderate risk |
| Torok et al. (2023) | Moderate risk: analysis was not controlled for potential confounders. | Low risk: the study used suicide data and did not involve participant selection. | Low risk: intervention status was well defined. | Low risk: no deviations from intended intervention. | Low risk: it is common that suicide number is underreported due to misclassification to other deaths. | Moderate risk: suicides occurred during interventions installation were not excluded in the analysis. | Low risk: results on the intervention site and other sites were reported in the same way. | Moderate risk |

**eTable 4. Suicide counts at intervention sites during pre- and post-intervention periods**

| **Author(s) and date** | **Duration of observation period (years)** | | **Total suicides** | | **Suicides per year** | |
| --- | --- | --- | --- | --- | --- | --- |
|  | **Pre-intervention** | **Post-intervention** | **Pre-intervention** | **Post-intervention** | **Pre-intervention** | **Post-intervention** |
| Beautrais (2001) | 4 | 4 | 15 | 3 | 3.8 | 0.8 |
| Bennewith et al. (2007) | 5 | 5 | 41 | 20 | 8.2 | 4.0 |
| Berman et al. (2022) | 6 | 30 | 17 | 5 | 2.8 | 0.2 |
| Clapperton et al. (2022) | 9.6 years per site on average | 3.0 years per site on average | 60 | 6 | 6.3 | 2.0 |
| Deisenhammer et al. (2024) | 10 | 10 | 26 | 9 | 2.6 | 0.9 |
| Dwyer et al. (2023) | 9.2 | 8.8 | 95 | 0 | 10.3 | 0.0 |
| Fredin-Knutzén et al. (2022) | 12 | 8 | 4 | 1 | 0.3 | 0.1 |
| Kõlves et al. (2023)^a^ | 15 | 6 | N/A | N/A | N/A | N/A |
| Law and Yip (2011) | 5 | 5 | 29 | 3 | 5.8 | 0.6 |
| Law et al. (2014) | 4 | 19 | 22 | 16 | 5.5 | 0.8 |
| Pelletier (2007) | 22.2 | 22.2 | 14 | 0 | 0.6 | 0.0 |
| Perron et al. (2013) | 13.5 | 5 | 146 | 13 | 10.8 | 2.6 |
| Reisch and Michel (2005) | 4 | 4 | 8 | 0 | 2.0 | 0.0 |
| Sinyor et al. (2017) | 11 | 11 | 105 | 1 | 9.5 | 0.1 |
| Skegg and Herbison (2009) | 10 | 2 | 13 | 0 | 1.3 | 0 |
| Torok et al. (2023) | 6 | 8 | 50 | 52 | 8.3 | 6.5 |
| **Total** | **131.5** | **145** | **645** | **129** | **78.2** | **18.6** |

^a^Suicide data at intervention site was not provided by the author due to small suicide number to avoid confidentiality issues.

Abbreviation: N/A, not available.

**eTable 5. Suicide counts at other sites during pre- and post-intervention periods**

| **Author(s) and date** | **Duration of observation period (years)** | | **Nearby site (same type)** | | | | **Other sites (same type)** | | | | **Other sites (different/unspecified type)** | | | |
| --- | --- | --- | --- | --- | --- | --- | --- | --- | --- | --- | --- | --- | --- | --- |
|  |  |  | **Total suicides** | | **Suicide per year** | | **Total suicides** | | **Suicide per year** | | **Total suicides** | | **Suicide per year** | |
|  | **Pre** | **Post** | **Pre** | **Post** | **Pre** | **Post** | **Pre** | **Post** | **Pre** | **Post** | **Pre** | **Post** | **Pre** | **Post** |
| Beautrais (2001) | 2 | 2 | N/A | N/A | N/A | N/A | N/A | N/A | N/A | N/A | 7 | 12 | 3.5 | 6.0 |
| Bennewith et al. (2007) | 5 | 5 | N/A | N/A | N/A | N/A | N/A | N/A | N/A | N/A | 31 | 42 | 6.2 | 8.4 |
| Berman et al. (2022) | 6 | 30 | 10 | 29 | 1.7 | 1.0 | 19 | 59 | 3.2 | 2.0 | N/A | N/A | N/A | N/A |
| Clapperton et al. (2022)^a^ | 9.6 years per site on average | 3.0 years per site on average | N/A | N/A | N/A | N/A | 57 | 16 | 5.9 | 5.3 | N/A | N/A | N/A | N/A |
| Deisenhammer et al. (2024) | 10 | 10 | 3 | 8 | 0.3 | 0.8 | 12 | 25 | 1.2 | 2.5 | 28 | 29 | 2.8 | 2.9 |
| Dwyer et al. (2023) | 9.2 | 8.8 | N/A | N/A | N/A | N/A | 43 | 62 | 4.7 | 7.0 | 93 | 143 | 10.1 | 16.3 |
| Fredin-Knutzén et al. (2022) | 12 | 8 | N/A | N/A | N/A | N/A | 10 | 14 | 0.8 | 1.8 | N/A | N/A | N/A | N/A |
| Kõlves et al. (2023) | 15 | 6 | N/A | N/A | N/A | N/A | 42 | 18 | 2.8 | 3.0 | 51 | 39 | 3.4 | 6.5 |
| Law and Yip (2011) | 5 | 5 | N/A | N/A | N/A | N/A | 22 | 20 | 4.4 | 4.0 | N/A | N/A | N/A | N/A |
| Law et al. (2014) | 4 | 19 | 15 | 73 | 3.8 | 3.8 | 6 | 14 | 1.5 | 0.7 | 13 | 118 | 3.3 | 6.2 |
| Pelletier (2007) | 22.2 | 22.2 | N/A | N/A | N/A | N/A | 1 | 2 | 0 | 0.1 | 2 | 1 | 0.1 | 0 |
| Perron et al. (2013) | 13.5 | 5 | N/A | N/A | N/A | N/A | 107 | 30 | 7.9 | 6.0 | 526 | 126 | 39.0 | 25.2 |
| Reisch and Michel (2005) | 4 | 4 | 6 | 3 | 1.5 | 0.8 | N/A | N/A | N/A | N/A | N/A | N/A | N/A | N/A |
| Sinyor et al. (2017) | 11 | 11 | 25 | 29 | 2.3 | 2.6 | 74 | 61 | 6.7 | 5.5 | 436 | 501 | 39.6 | 45.5 |
| Skegg and Herbison (2009) | 10 | 2 | N/A | N/A | N/A | N/A | 4 | 0 | 0.4 | 0 | N/A | N/A | N/A | N/A |
| Torok et al. (2023) | 6 | 8 | 12 | 13 | 2.0 | 1.6 | N/A | N/A | N/A | N/A | 10 | 15 | 1.7 | 1.9 |
| **Total** |  |  | **71** | **155** | **11.6** | **10.6** | **397** | **321** | **39.5** | **37.9** | **1,197** | **1,026** | **109.7** | **118.9** |

^a^Only data for 500m radius (not 1000m radius) were used in our analysis.

Abbreviation: N/A, not available.

**eTable 6. Counts of suicides by the same method as that used at the intervention site, and by other methods, and all suicides during pre- and post-intervention periods**

| **Author(s) and date** | **Duration of observation period (years)** | | **Suicides by the same method** | | | | **Suicides by other methods** | | | | **All suicides** | | | |
| --- | --- | --- | --- | --- | --- | --- | --- | --- | --- | --- | --- | --- | --- | --- |
|  |  |  | **Total suicides** | | **Suicide per year** | | **Total suicides** | | **Suicide per year** | | **Total suicides** | | **Suicide per year** | |
|  | **Pre** | **Post** | **Pre** | **Post** | **Pre** | **Post** | **Pre** | **Post** | **Pre** | **Post** | **Pre** | **Post** | **Pre** | **Post** |
| Beautrais (2001) | 2 | 2 | 14 | 14 | 7.0 | 7.0 | N/A | N/A | N/A | N/A | N/A | N/A | N/A | N/A |
| Bennewith et al. (2007) | 5 | 5 | 72 | 62 | 14.4 | 12.4 | N/A | N/A | N/A | N/A | N/A | N/A | N/A | N/A |
| Berman et al. (2022) | 6 | 30 | N/A | N/A | N/A | N/A | N/A | N/A | N/A | N/A | 306 | 1,086 | 51.0 | 36.2 |
| Deisenhammer et al. (2024) | 10 | 10 | 167 | 154 | 16.7 | 15.4 | N/A | N/A | N/A | N/A | N/A | N/A | N/A | N/A |
| Dwyer et al. (2023) | 9.2 | 8.8 | 231 | 205 | 25.1 | 23.3 | 4,831 | 5,476 | 525.1 | 622.3 | 5,062 | 5,681 | 550.2 | 645.6 |
| Law and Yip (2011) | 5 | 5 | 51 | 23 | 10.2 | 4.6 | 4,393 | 5,081 | 878.6 | 1,016.2 | 4,444 | 5,104 | 888.8 | 1,020.8 |
| Law et al. (2014) | 4 | 19 | 56 | 221 | 14.0 | 11.6 | 701 | 4,254 | 175.3 | 223.9 | 757 | 4,475 | 189.3 | 235.5 |
| Pelletier (2007) | 22.2 | 22.2 | 17 | 3 | 0.8 | 0.1 | N/A | N/A | N/A | N/A | N/A | N/A | N/A | N/A |
| Perron et al. (2013) | 13.5 | 5 | 779 | 169 | 57.7 | 33.8 | 18,759 | 5,391 | 1,389.6 | 1,078.2 | 19,538 | 5,560 | 1,447.3 | 1,112.0 |
| Reisch and Michel (2005) | 4 | 4 | 45 | 44 | 11.3 | 11.0 | N/A | N/A | N/A | N/A | N/A | N/A | N/A | N/A |
| Sinyor et al. (2017) | 11 | 11 | 652 | 624 | 59.3 | 56.7 | 2,175 | 1,952 | 197.7 | 177.5 | 2,827 | 2,576 | 257.0 | 234.2 |
| Skegg and Herbison (2009) | 10 | 2 | 17 | 0 | 1.7 | 0 | 132 | 29 | 13.2 | 14.5 | 149 | 29 | 14.9 | 14.5 |
| Torok et al. (2023) | 6 | 8 | 40 | 54 | 6.7 | 6.8 | N/A | N/A | N/A | N/A | N/A | N/A | N/A | N/A |
| **Total** |  |  | **2,141** | **1,573** | **224.9** | **182.7** | **30,991** | **22,183** | **3,179.5** | **3,132.6** | **33,083** | **24,511** | **3,398.5** | **3,298.8** |

Abbreviation: N/A, not available.


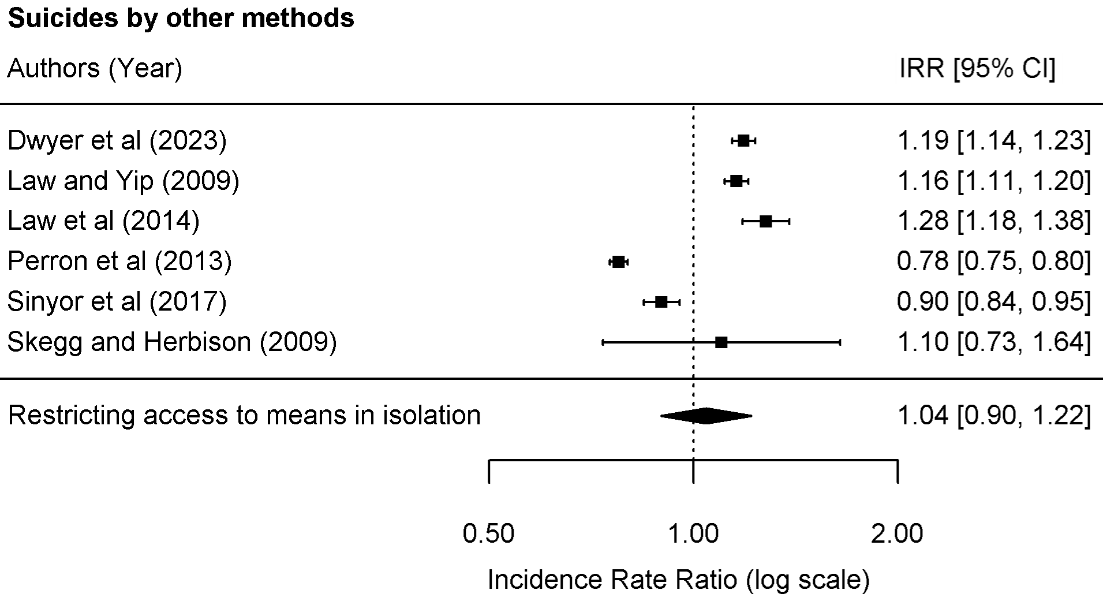


**eFigure 1. Risk of suicide by other methods after deploying intervention that restricts access to means at frequently used suicide location.**


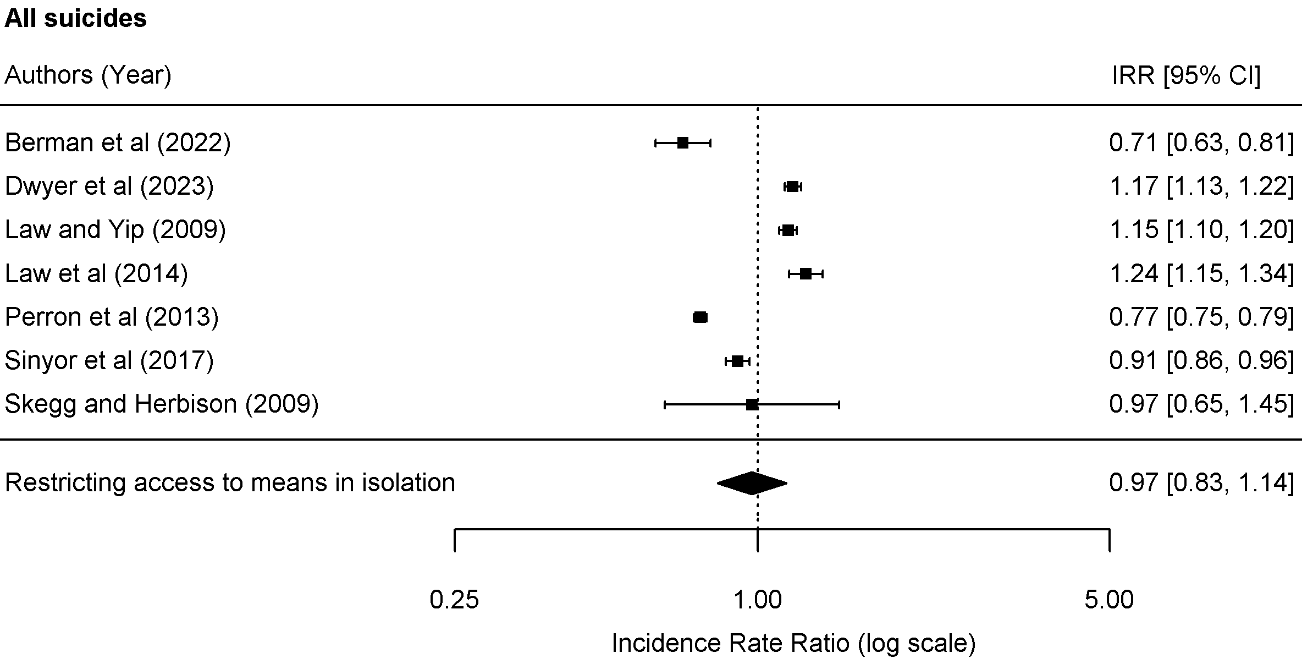


**eFigure 2. Risk of suicide after deploying intervention that restricts access to means at frequently used suicide location.**


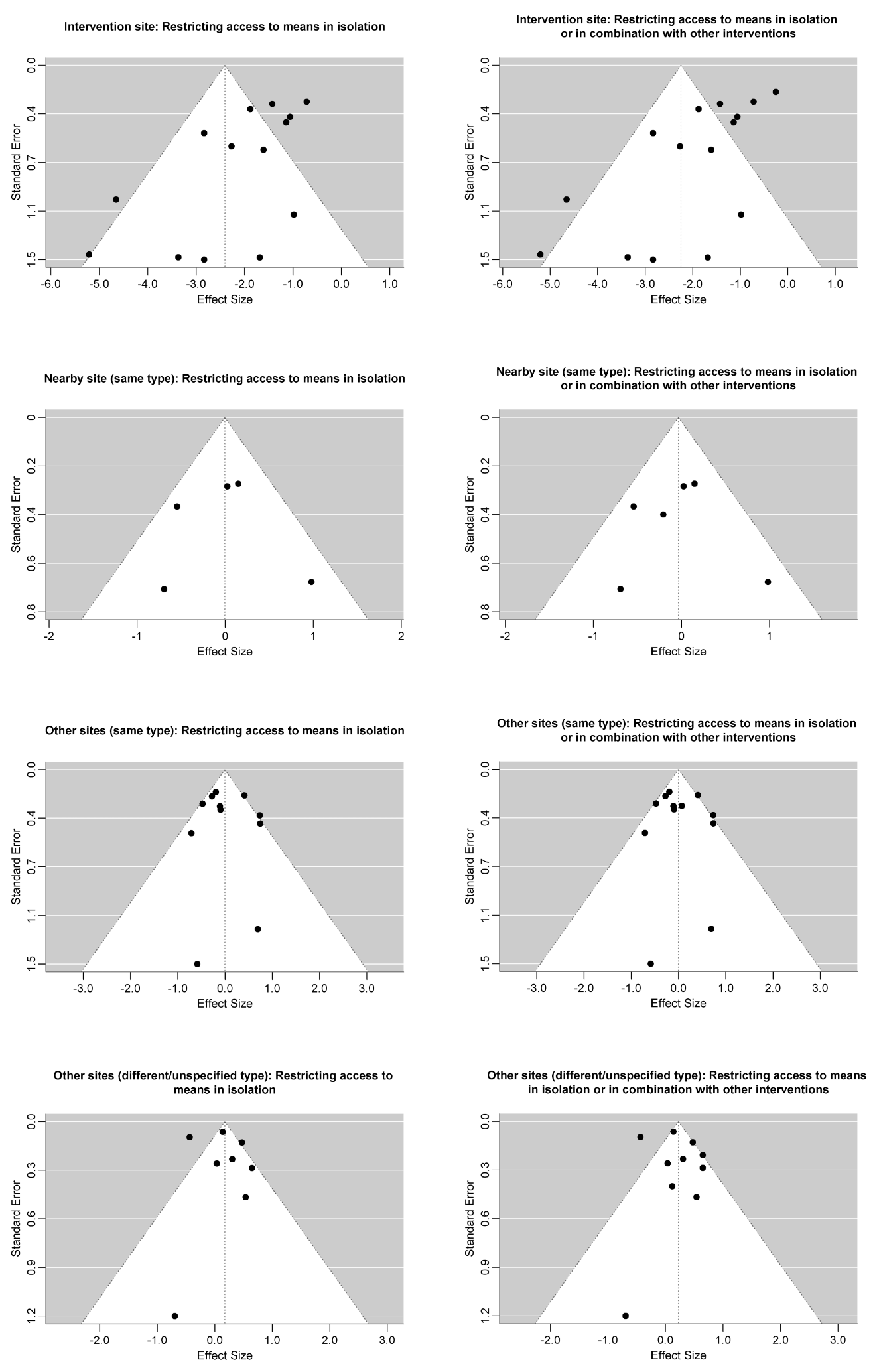


**eFigure 3. Funnel plots for the models of intervention and other sites.**


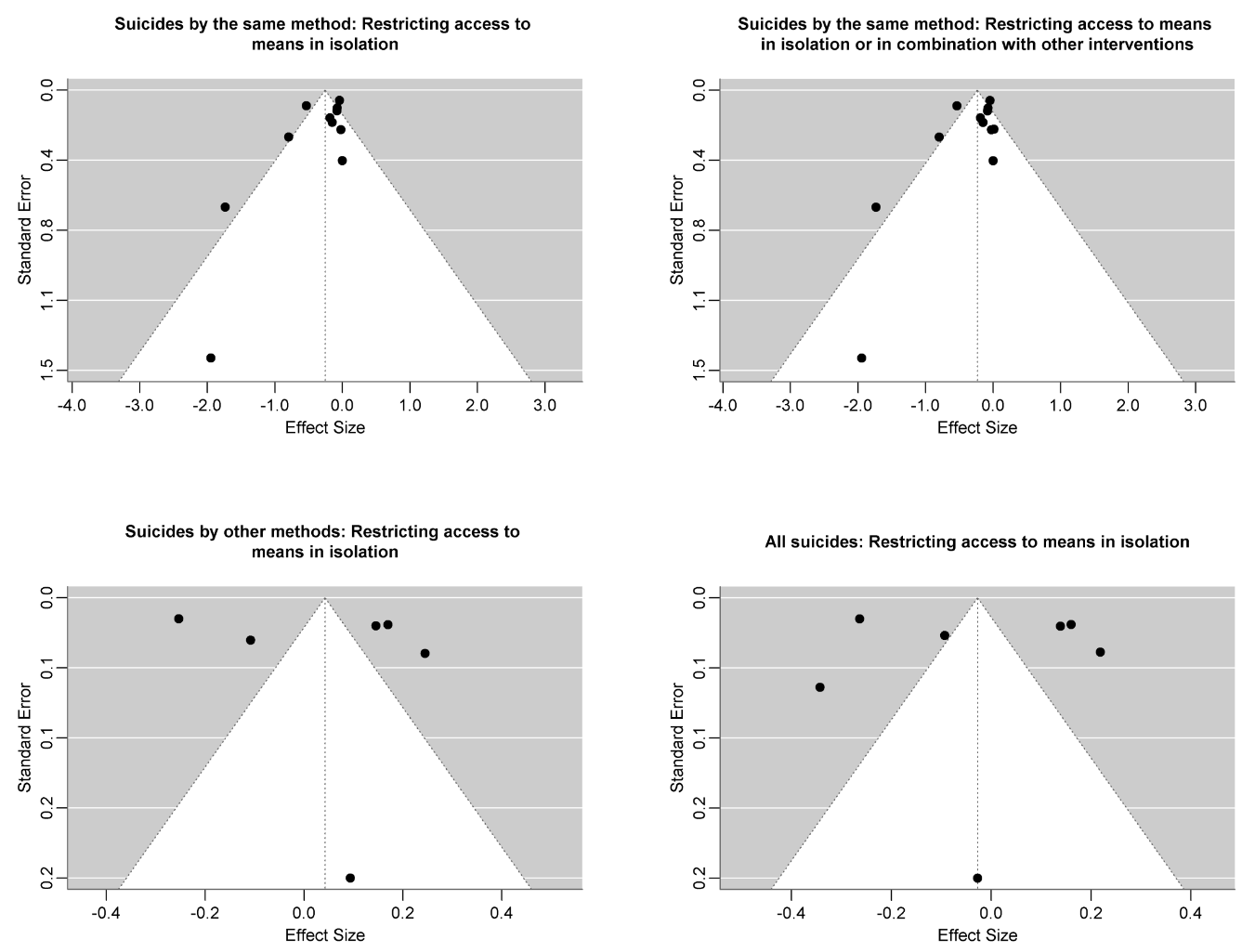


**eFigure 4. Funnel plots for the models of suicides by the same method and other methods, and all suicides.**
